# The Training and Development of Elite Sprint Performance: an Integration of Scientific and Best Practice Literature

**DOI:** 10.1186/s40798-019-0221-0

**Published:** 2019-11-21

**Authors:** Thomas Haugen, Stephen Seiler, Øyvind Sandbakk, Espen Tønnessen

**Affiliations:** 1 Faculty of Health Sciences, Kristiania University College, PB 1190 Sentrum, 0107 Oslo, Norway; 2 Faculty of Health and Sport Sciences, University of Agder, PB 422, 4604 Kristiansand, Norway; 3 Centre for Elite Sports Research, Department of Neuromedicine and Movement Science, Norwegian University of Science and Technology, 7491 Trondheim, Norway

**Keywords:** Sprint conditioning, Training guidelines, Training volume, Acceleration, Maximal velocity

## Abstract

Despite a voluminous body of research devoted to sprint training, our understanding of the training process leading to a world-class sprint performance is limited. The objective of this review is to integrate scientific and best practice literature regarding the training and development of elite sprint performance. Sprint performance is heavily dependent upon genetic traits, and the annual within-athlete performance differences are lower than the typical variation, the smallest worthwhile change, and the influence of external conditions such as wind, monitoring methodologies, etc. Still, key underlying determinants (e.g., power, technique, and sprint-specific endurance) are trainable. In this review, we describe how well-known training principles (progression, specificity, variation/periodization, and individualization) and varying training methods (e.g., sprinting/running, technical training, strength/power, plyometric training) are used in a sprint training context. Indeed, there is a considerable gap between science and best practice in how training principles and methods are applied. While the vast majority of sprint-related studies are performed on young team sport athletes and focus on brief sprints with maximal intensity and short recoveries, elite sprinters perform sprinting/running over a broad range of distances and with varying intensity and recovery periods. Within best practice, there is a stronger link between choice of training component (i.e., modality, duration, intensity, recovery, session rate) and the intended purpose of the training session compared with the “one-size-fits-all” approach in scientific literature. This review provides a point of departure for scientists and practitioners regarding the training and development of elite sprint performance and can serve as a position statement for outlining state-of-the-art sprint training recommendations and for generation of new hypotheses to be tested in future research.

## Key Points

- There are considerable gaps between science and best practice in how training principles and training methods should be applied for elite sprint performance
- This review serves as a position statement for outlining state-of-the-art sprint training recommendations
- We provide a point of departure for discussion between scientists and practitioners regarding the training and development of sprint performance

## Background

The crowning of the 100-m sprint champion remains a hallmark of each Olympic Games, and the winners are “the world’s fastest humans.” The dramatic world record progression since the first modern Olympics has been driven by advancing training methodology and deliberate practice, combined with key improvements in running surfaces and footwear. Because sprint running is a core capacity that underlies performance in many sports, there is a voluminous body of scientific literature devoted to sprint training. The vast majority of sprint-related training interventions have reported positive effects on sprinting capabilities [1–4], leading to the assumption that sprinting performance is easily improved with a variety of methods. In contrast, observations of elite athletes over time show a very different reality, one where most annual within-athlete performance differences are lower than typical variation, the smallest worthwhile change, and the influence of external conditions (wind, temperature, altitude, timing methods/procedures, etc.) [5, 6]. Plausible explanations for this mismatch between published science and observed practice are (1) publication bias in favor of positive findings and (2) subject training status bias, with most experimental data coming from studies of untrained or moderately trained performers.

In contrast to the many descriptive studies of world-class endurance athletes (e.g., [7–9]), no studies of world-class sprinters to date have described the varying training components (modality, duration, intensity, resting periods, session rate, etc.) across the annual cycle. It is fair to say that positive developments in sprint training methods employed by world-class athletes have not been driven by sports scientists. Publicly available “recipe books” and training guides based upon the practical experience and intuition of world-leading sprint coaches, and also governing body documents from acknowledged athletics federations, have become important and popular sources of best practice training information and framework development for the international sprint community [10–18]. We believe combining data sources from available research evidence and results-proven practice provides a valid point of departure for outlining state-of-the-art sprint training recommendations and for generation of new hypotheses to be tested in future research. The objective of this review is therefore to integrate scientific and best practice literature regarding the training and development of elite sprint performance. Although the present review is anchored in athletics and competitive 100-m sprinting, most of the content is also relevant for other sports where linear sprints frequently occur.

## Sprint Performance Determinants

The 100-m sprint has traditionally been categorized into three main phases: acceleration, maximal velocity, and deceleration [19, 20]. The acceleration phase can in turn be segregated into initial (start block and reaction), middle, and final subsections [21]. Reaction time in world-class sprinters is typically 0.17–0.18 ± 0.03 s [22]. The shape of the velocity curve is consistent across performance level, but the duration and quality of each phase vary from athlete to athlete. Overall, maximal velocity is highly correlated with 100-m sprint performance, and the best sprinters accelerate over a longer distance than their lower performing counterparts [23, 24]. Table 1 displays an overview of observed split times as a function of 100-m sprint performance level [25–32] and can be used to identify individual strengths and weaknesses across the varying phases.

**Table 1:** Split times (mean ± SD) across 100-m sprint performance level

	*n*	30 m (s)	60 m (s)	80 m (s)	30–60 m (s)	60–80 m (s)	80–100 m (s)	60–100 m (s)
100 m men								
9.58^a^	1	3.78	6.31	7.92	2.53	1.61	1.66	3.27
9.71–9.80	5	3.82 ± 0.01	6.37 ± 0.03	8.05 ± 0.03	2.55 ± 0.02	1.68 ± 0.03	1.72 ± 0.03	3.40 ± 0.05
9.81–9.90	7	3.83 ± 0.04	6.42 ± 0.04	8.12 ± 0.02	2.60 ± 0.02	1.70 ± 0.03	1.75 ± 0.02	3.45 ± 0.05
9.91–10.00	12	3.85 ± 0.04	6.46 ± 0.04	8.18 ± 0.04	2.61 ± 0.02	1.73 ± 0.01	1.77 ± 0.02	3.50 ± 0.03
10.01–10.10	21	3.89 ± 0.04	6.51 ± 0.03	8.26 ± 0.03	2.62 ± 0.03	1.74 ± 0.01	1.79 ± 0.02	3.54 ± 0.04
10.11–10.20	24	3.95 ± 0.04	6.60 ± 0.05	8.36 ± 0.05	2.64 ± 0.03	1.75 ± 0.01	1.81 ± 0.04	3.57 ± 0.03
100 m women								
10.61–10.70	4	4.07 ± 0.02	6.89 ± 0.03	8.76 ± 0.03	2.82 ± 0.01	1.87 ± 0.02	1.90 ± 0.05	3.78 ± 0.06
10.71–10.80	12	4.10 ± 0.03	6.94 ± 0.04	8.83 ± 0.03	2.84 ± 0.01	1.88 ± 0.02	1.93 ± 0.03	3.82 ± 0.04
10.81–10.90	5	4.17 ± 0.06	6.99 ± 0.04	8.89 ± 0.03	2.85 ± 0.02	1.90 ± 0.01	1.95 ± 0.03	3.86 ± 0.04
10.91–11.00	6	4.17 ± 0.07	7.05 ± 0.04	8.97 ± 0.03	2.87 ± 0.04	1.92 ± 0.02	1.99 ± 0.03	3.92 ± 0.04
11.01–11.10	6	4.18 ± 0.05	7.09 ± 0.03	9.03 ± 0.02	2.89 ± 0.04	1.94 ± 0.02	2.01 ± 0.02	3.95 ± 0.03
11.11–11.20	9	4.20 ± 0.05	7.15 ± 0.05	9.13 ± 0.04	2.95 ± 0.02	1.98 ± 0.02	2.04 ± 0.04	4.02 ± 0.05

The calculations are based on biomechanical reports from international championships [23–30]

^a^Usain Bolt’s world record race from 2009

Power, technique, and sprint-specific endurance are considered key underlying determinants of 100-m sprint performance [3, 11–16, 24, 33, 34]. A very strong relationship exists between maximal horizontal power output and sprint performance; the shorter the sprint distance is, the higher the association with maximal horizontal power output [35]. Power output demand in sprinting increases exponentially with velocity [35, 36]. Slawinski et al. [23] reported that step averaged maximal horizontal power output in male and female world-class sprinters was 30.3 ± 2.5 and 24.5 ± 4.2 W kg^− 1^, respectively, typically reached after ~ 1 s of sprinting. The highest individual values for men and women were 36.1 and 29.3 W kg^− 1^ respectively, representing current upper limits in humans [37].

Although the basic principles of sprinting are relatively simple and governed by the laws of motion, the way an athlete solves the mechanical constraints and utilizes the degrees of freedom within these constraints is far more complex [24]. A review of research literature shows that the following kinematic variables have received the most attention [38–47]:

- Spatiotemporal variables (e.g., step length, step rate, contact time, flight/aerial time)
- Segment configuration at touchdown and lift-off
- Lower-limb segment velocities immediately prior to touchdown or during ground contact
- Front- and back-side mechanics

Indeed, sprint mechanical variables are entangled, and no single variable is associated with better performance [24]. Because kinetics and kinematics are entwined, athletes cannot apply sprinting mechanics that they are not adequately predisposed to. For more information regarding the sprint running technique, we refer to previously published biomechanical analyses (e.g., [20, 24, 33, 34, 38–51]).

Sprint-specific endurance refers to the deceleration phase of the sprint. The velocity decline is typically accompanied by a reduction in step rate [24]. Sprint-related fatigue is attributed to disturbances in the central nervous system and peripheral factors within the skeletal muscles [52–55]. Available research indicates that leg stiffness, which influences elastic energy storage, is particularly crucial for sprint-specific endurance [56–61]. Sprint-specific endurance is also determined by instantaneous energy delivery [24]. Estimated from accumulated oxygen deficit measures, the relative anaerobic energy system contribution (from stored adenosine triphosphate, stored phosphocreatine, and anaerobic glycolysis) is about 80% for 100-m sprint [62].

Sprint performance is heavily dependent upon genetic traits. Charlie Francis, an acknowledged sprint coach, stated that “sprinters are born, not made” [14]. However, which genetic profiles make the greatest contribution has been the subject for much debate [63, 64], as genetic predispositions include not only anthropometric characteristics and muscle fiber-type proportions, but also the capacity to adapt to training [65, 66]. In general, elite performance appears polygenetic, with the contribution of numerous genetic variants being additive.

## Sprint Performance Development

Sprint performance capacity evolves and devolves throughout life via growth, maturation, training, and aging [5, 67–69]. Age of peak performance in world-class sprinters is typically 25–26 years [5, 70, 71]. However, the concept of training age needs to be considered when assessing age of peak performance. Athletes who start with specialized training at a young age may also tend to reach their peak performance at an earlier age than their counterparts who specialize somewhat later.

Haugen et al. [5] reported that for world top 100 sprinters in their early 20s, mean annual improvements were in the range of only 0.1–0.2%. The very best athletes generally display greater improvement in the years just preceding age of peak performance compared with their lower performing counterparts [5, 72, 73]. For example, Haugen et al. [72] reported that the world’s all-time best male and female sprinters improved by an average of 8% from 18 years of age, while the corresponding improvement for Norwegian national-level competitive sprinters was 1.3–1.4%. The potential use of doping among some of the investigated athletes may have affected these results, but trainability variations across performance level may also be explained by other factors (e.g., training status, responsiveness to training, coaching quality, nutrition, etc.). Nevertheless, it becomes very challenging to enhance or even maintain sprint performance beyond the age of 30 [5, 70], most likely due to neural and/or hormonal factors and an age-related decrease in type II fiber distribution and/or cross-sectional area [74–76].

Identification and development of sprint talent are confounded by the observed variation in physical maturation rate and timing. Elite junior athletics is characterized by a combination of marked relative-age effects and high attrition rates. Many young athletes may be discouraged from continuing to senior level before realizing their full potential [77, 78]. Boccia et al. [79] observed that, within a group of talented young performers, athletic performance in the early teens is not a good predictor of senior performance in long and high jump. It is reasonable to assume that this is also true for sprinting.

Taken all findings together, sprinters who perform at a high junior level without excessive specialization are at the optimal point of departure for senior success. Excessive specialization and inappropriate training progression increase the odds of nonfunctional overreaching, overtraining, and performance stagnation [80, 81]. A widely held view is that elite performance requires ~ 10 years or 10,000 h of deliberate practice to acquire the necessary skills and experience to perform at an international level [82, 83]. Although the deliberate practice framework has gained popularity in sport science and in popular literature, its applicability to sprint running is very limited. The publicly available biography of Usain Bolt [84] exemplifies that extremely talented athletes can reach an international level within only 5–6 years of deliberate practice. Indeed, there is considerable variation among athletes and numerous routes to expertise under optimal conditions. As a foundation for long-term training strategy, coaches rely on well-established training principles to design programs and make educated decisions.

## Training Principles

### Progressive Overload

Long-term performance development is only achieved when athletes are exposed to a systematic increase in training load over time, while adequate recovery is ensured [85]. Indeed, the capacity to perform and absorb large training loads is seen as both an adaptation over time and a talent in itself. Training load in sprint running is determined by a series of components such as training modality (e.g., sprinting/running, strength training, plyometric training), duration, intensity, resting periods, session rate, running surface, and footwear [10–16]. These components will be treated more in detail in a sprint-specific setting later in this review.

The principle of progressive overload is envisioned to reduce the risk of injury and overtraining while stimulating long-term training adaptations. Excessive and rapid increases in training loads are likely responsible for a large proportion of soft-tissue injuries [86, 87]. In sprinters, the training phase immediately following the off-season and the transition phase between the preparation period and competitive season are particularly vulnerable periods for injury. Haugen et al. [88] observed that two-thirds of all hamstring injuries in competitive sprinters occurred in the transition period between specific preparation and competition season. This period is ideally characterized by large reductions in training volume, increases in training intensity/sprint speed, and positive “bumps” in individual sprint performance development. Therefore, during the initial weeks of a sprint training program, there should be a gradual familiarization, both in terms of intensity and duration/repetitions. Moreover, it is crucial that sprinters gradually mobilize their maximal sprinting capacity as the competitive season approaches. When the difference between training speed and competition speed is too large, injury risk appears to increase, but aggregated data on this relationship in competitive sprinters are lacking.

Running surface and footwear are crucial and specific modifiers of training load for sprinting. It is generally assumed that the harder the surface is, the higher the neuromuscular load for the lower limbs [10, 11, 13–15]. Most elite sprinters perform high-intensive sprinting sessions with spike shoes on a rubberized track surface. Because such training is demanding for the central nervous system, empirical evidence suggests that intensive sprinting sessions require at least 48 h of recovery. Hence, sprinting on consecutive days rarely occurs among leading practitioners [10–18]. In contrast, recovery sessions or low-intensive intervals are typically performed with cushioned running shoes/trainers on grass or artificial turf. There is a long tradition for low-intensive interval training on grass in Jamaican sprinting [10].

### Specificity

Training adaptations are specific to the stimulus applied, encompassing movement patterns and force-velocity characteristics such as muscle actions and muscle groups used, speed of movement, range of motion, training load, and energy systems involved [89]. Based on these considerations, it is not surprising that sprint running and high-velocity movements are paramount for sprint performance enhancement [4, 90]. According to Charlie Francis, the main stimulus is the number of sprinting meters at high intensity [13, 14]. However, there are also variations within specific conditions. For example, sprint running can be performed under assisted or resisted conditions. Other “less specific” training forms such as strength, power, and plyometric training are commonly performed to target the underlying components of sprint performance [10–18]. Although these training forms do not duplicate the holistic sprint running movement, they provide targeted stimuli of important components that limit sprint performance. The varying training methods for sprint performance enhancements are treated more in detail later in this review.

### Variation and Periodization

The principle of variation builds on the notion that systematic variation in specific training variables is most effective for long-term adaptations [90–92]. According to the American College of Sports Medicine (ACSM), advanced athletes should perform training with higher relative loading in a periodized fashion. The higher the performance level, the more systematic variation is recommended [90]. The most commonly investigated training theory involving planned training variation is periodization, an often misused term that today refers to any form of training plan, regardless of structure [92].

Matveyev was the first to write a book about training periodization in the 1960s [93]. A key feature for the traditional periodization model was early emphasis on high training volume with low intensity, followed by a gradual transition to higher training intensity and reduced volume as the competition periods approached. Several leading sprint coaches are skeptical of the classic periodization model because (1) the initial high-volume/low-intensity training leads to inappropriate adaptations, (2) the high-intensity training closer to the competition season encompasses insufficient volume, and (3) the steep intensification of training at the end of the preparation period leads to an unnecessary increase in injury risk [11, 13, 14]. It should be noted, however, that many sprint coaches apply a form of traditional periodization, although with fewer fluctuations in intensity and volume than Matveyev’s original model.

Block training periodization was introduced by Verkhoshansky [94] in the 1980s and has been widely used by prominent coaches. The term “block training” has generally been understood to consist of training cycles of highly concentrated specialized workloads. The usefulness of block training has also been questioned by acknowledged sprint coaches, as the model prohibits developed skills to be maintained throughout the varying meso-cycles [11]. Publicly available information indicates that alternative periodization models are used within elite sprinting communities [10–16]. Choice of periodization model seems to depend on sprint distance (100 m vs. 400 m), daily situation (high school/college/university level, professionals vs. semi-professionals or amateurs), and tradition. Some coaches classify the training year into one preparation phase and one competition period. Double periodization (i.e., two peaking phases) is more common, consisting of a preparation phase, an indoor season, a new preparation phase, and finally an outdoor competition season. Some of the very best athletes also split the outdoor season into early and late peaks in order to prepare for national trials and international championships. Classification of training into “heavy” and “easy” weeks within the preparation periods is another important aspect within periodization. Leading practitioners typically use a 2:1 or 3:1 periodization, that is, 2 or 3 weeks with relatively high training load are followed by an easier training week for recovery purposes [10, 11, 13, 14].

The “long-to-short” periodization model is typically applied by long-sprint specialists [11]. This approach focuses on long distances in the early preparation period and progresses to short distances throughout the training year [11, 15, 16]. An even more popular periodization model for sprinters was introduced by Charlie Francis in the 1980s, termed “short to long” [13, 14]. Here, training periods are mainly differentiated by the relative emphasis on each phase of a sprint: acceleration, maximum velocity, and deceleration. The initial meso-cycles focus on short sprints and power training, culminating with the indoor season where 60 m is the main event. Maximal velocity is prioritized after the indoor season, while sprint-specific endurance becomes more prioritized when approaching the outdoor season. According to the model, it is easier to improve maximal velocity and then extend the duration that velocity can be maintained. The short-to-long periodization model ensures that developed skills are not lost. Maintaining key elements while adjusting the demand of a given skill is a vital principle. This short-to-long approach has been used by numerous leading practitioners in the last decades [10, 11, 13–16].

Another key feature within the short-to-long periodization model is the polarized training concept. More specifically, sprinting intensity should be either ≥ 95% or < 70% of maximal velocity to enhance performance or facilitate recovery, respectively [11, 13, 14]. It is assumed that mid-range intensities (~ 70–95%) are not beneficial either for performance or recovery and should be avoided. Dan Pfaff, the coach of Donovan Bailey (former 100-m world record holder and Olympic Champion), has for many years practiced a concurrent, polarized, and short-to-long model of thinking within the micro-cycle build approaching a competition season [11, 17]. Three-day training blocks are utilized: short accelerations are performed on Monday, maximal velocity sprinting on Wednesday, and sprint-specific endurance on Friday. We note that the polarized training organization performed in certain sprinting communities bears great resemblance with training intensity distribution in elite endurance athletes, which is typically organized after a polarized pattern (e.g., [7, 95, 96]). In sprint running, the polarized approach might resemble required training quality when training at the highest velocities, and at the same time sufficient volume of sprinting. Training in between these “zones/velocities” might be “a black hole”, where neither the quality nor the quantity for further development is achieved. Overall, the physiological mechanisms underlying the polarized training concept are far from understood, and future studies should pay more attention to this topic.

In conclusion, elite coaches plan the training of their athletes with significant detail. However, the underlying mechanisms for the superiority of specific periodization models in sprint running remain unclear, and there is no direct evidence enabling us to compare outcomes across the various periodization methodologies [92].

### Individualization

Individualization is a general training principle and refers to the idea that training must be prescribed according to individual performance capacity and predispositions such as anthropometric factors, training status/age, sex, recovery/injury status, and force-velocity profiles [10, 13, 14, 90, 97]. For example, the kinematics of sprinting varies according to performance level and anthropometric factors. This includes spatiotemporal variables, start block positioning, trunk angle during the early acceleration phase, and lower limb joint angles [20, 38, 40, 51]. Hence, coaches cannot implement sprinting mechanics that their athletes are not predisposed to by nature and prepared for through training. For example, a mediocre athlete will likely sprint slower when trying to adapt the step length of a world-class sprinter as the ground reaction forces typically become more vertically oriented.

Total training volume generally increases with training age, but as athletes approach their maximum potential at the end of their career, training volume may decrease to accommodate increased need for recovery time between high-intensity sessions [15, 16]. This coincides well with age-related changes in anabolic hormone concentration that play a crucial role in the body’s metabolic, tissue repair, and anabolic capabilities in response to training. For example, testosterone is positive for sprint performance [98]. From the third decade, circulating testosterone levels decline gradually each year [74, 99]. There are also sex differences in endocrine response to training, as several strength training studies have revealed significant elevations in the recovery of testosterone and free testosterone in men through 30 min into recovery, while no or limited acute elevations have been observed in women [100]. Within this context, it is interesting to note that Stephen Francis, an acknowledged Jamaican sprint coach, has argued that women should perform training sessions with 20% less volume than men [10]. A counter argument would be that women are able to absorb higher training volumes, because their maximal velocity is ~ 10% lower than men (corresponding to substantially lower peak force and power loads on the neuromuscular apparatus). The scientific training literature provides very limited information regarding potential sex differentiation of training prescription, and future studies should devote more attention to this topic.

Training history appears to modulate recovery processes, but this interplay is not well appreciated in the research literature. In the American College of Sports Medicine position stand, the recommendations for rest period length and training frequency for power training are like those for novice, intermediate, and advanced athletes [90]. In contrast, the guidelines outlined by the UK Athletics state that duration, number of repetitions, and recovery time in sprint-specific training sessions should be adjusted according to training status and performance level [15, 16]. For example, an underlying assumption in high-performance environments is that each sprint performed by an elite athlete is more demanding on the entire neuromuscular system than for their lower performing counterparts, and hence, more recovery time between each sprint is needed [15, 16]. Future research should aim to verify this claim.

It has recently been suggested that individualized sprint training should be based on force-velocity (Fv) profiles [97, 101, 102]. A possible avenue for such an approach is individual test comparison with group mean values, where athletes with velocity deficits should be prescribed more maximal velocity sprinting, while athletes with horizontal force deficits should prioritize more horizontal strength work [97]. Although reference values have been outlined for athletes across sprint performance levels [23, 35, 38], it remains unclear if such an approach is effective [103]. The logic of this approach builds on an assumed direct relationship between acceleration and peak velocity measurements for the runner and the underlying contractile characteristics of the muscle groups involved. However, the fascicle shortening velocities of active muscles do not necessarily change with increasing running velocity [104–106]. The relationship between changes in running velocity and muscle fascicle shortening velocity appears to be complicated by an increased contribution from elastic properties with increasing running velocity [104–106]. Running velocity is not a proxy for muscle contraction velocity, and for this reason, Helland et al. [107] have questioned the use of Fv profiling in this context. More research is required regarding how training should be evaluated and modified based on force-velocity assessments.

## Training Methods

### Sprint Training

The vast majority of scientific studies investigating sprint training methods are performed on young team sport athletes where brief sprints with short recoveries are the norm [1–4]. Therefore, sprint training recommendations from the research literature have limited relevance to competitive sprinting, where elite 100-m athletes perform sprint-specific training over various distances. Practitioners classify sprint running either according to phase of interest or primary energy system used [11–16]. For the latter, sprint duration shorter than 6–7 s is considered alactic, while longer sprints are considered lactic [11–16]. In the following paragraphs, we present best practice guidelines for specific sprint training according to phase of interest. Total volume within these sessions is typically guided by the intensity and visual inspection of technique. That is, the session should be ended when drop-off in performance and/or technical deterioration is observed [11, 13–16]. Table 2 summarizes the best practice guidelines, while Table 3 shows examples of training weeks across varying meso-cycles.

**Table 2:** Summary of best practice sprint training recommendations

Training method	Distance (m)	Intensity (%)	Recoveries (min)	Total session volume (m)	Initiation	Time to next HIS (hours)	Footwear and surface
Acceleration	10–50	> 98	2–7	100–300	Block/3-point/crouched	48	Spikes on track
Maximal velocity	10–30^a^	> 98	4–15	50–150^a^	20–40-m flying start	48–72	Spikes on track
Sprint-specific endurance	80–150	> 95	8–30	300–900	Standing start	48–72	Spikes on track
Speed endurance	60–80	90–95	2–4 (8–15)	600–2000	Standing start	48–72	Spikes on track
Resisted sprints	10–30	80–95^b^	3–6	50–200	3-point/crouched	48	Optional
Assisted sprints	10–30^a^	≤ 105	5–15	≤ 100^a^	20–40-m flying start	48	Spikes on track
Tempo	100–300	60–70	1–3	1000–2000	Standing start	24	Trainers on grass

Intensity is expressed in percent of maximal velocity. Recovery = time between repetitions (sets). *HIS* = high-intensive session

^a^Flying start distance excluded

^b^The perceived effort is maximal, so the velocity decline is caused by resistance loading

**Table 3:** Training week examples across varying meso-cycles

Day	Early preparation period	Mid-preparation period	Late preparation period	Mid-season
Mon	Hill sprints	Resisted sprints	Acceleration	Acceleration and maximal velocity
Tue	Hypertrophy strength	Maximal strength	Explosive strength + plyometrics	Plyometrics
Wed	Tempo	Tempo	Tempo	Tempo
Thu	Speed endurance	Speed endurance	Maximal velocity	Sprint-specific endurance
Fri	Hypertrophy strength	Maximal strength	Explosive strength + plyometrics	Plyometrics
Sat	Tempo	Tempo	Tempo	Tempo
Sun	Off	Off	Off	Off

See Table 2 for the session-specific training content

#### Acceleration

When acceleration is the primary focus, leading practitioners recommend 10–50-m sprints from blocks, crouched or a three-point start position [10, 11, 13–18]. Block starts are considered more energetically costly than standing starts. The distances used will vary depending on athlete performance level, as better sprinters reach higher top speeds and accelerate longer than their lower performing counterparts. Full recovery is required between each sprint, allowing the athlete to perform each repetition without a drop-off in performance. According to the UK Athletics, longer recoveries are required for elite sprinters who are reaching higher absolute intensities than for younger developmental athletes [15]. A typical acceleration session for a young and relatively untrained athlete might be runs over 20 m from a crouched start with 2-min recovery between each repetition, while an elite sprinter may perform sprints over 40 m from blocks with 7-min recovery in between [15].

#### Maximal Velocity

Flying sprints are typically recommended when the focus is to develop maximal velocity [11, 13–16]. The aim is to reach the highest velocity possible and continue the sprint run for only as long as velocity does not decrease. Athletes are able to maintain maximal velocity for only around 10–30 m, depending on performance level and training status [31, 32]. Flying sprints are often performed from a rolling (jog in) start. Although the rate of acceleration is reduced, the athlete may be able to achieve a higher maximum velocity or reach the same velocity as after maximal acceleration but using less energy. The run-up distance typically ranges from 20 to 60 m, depending on the distance an athlete needs to reach the highest speeds. Young and relatively untrained athletes may use a 20-m build-up for 10-m flying sprints with ~ 4-min recovery in between. In contrast, elite competitors may use a 40-m build-up for 30-m flying sprints. Because their speeds may approach 12 m s^− 1^, the recovery interval may need to be ~ 15 min before they can reproduce the performance again [11, 13–16].

#### Sprint-Specific Endurance

The aim of sprint-specific endurance training is to improve the ability to maintain sprint velocity for as long as possible. Such training is typified by runs lasting 7–15 s at 95–100% intensity, with full recovery used between repetitions and sets [11, 13–18]. A rule of thumb among practitioners is that 1–2-min recovery is required for every second spent on maximal sprinting [15, 16]. The higher the performance standard, the longer the recovery periods are required. While 2–3 × 100-m sprints with 10-min recovery may be an adequate sprint-specific endurance session for a relatively untrained junior, a well-trained elite competitor may perform 4–6 × 150-m sprints with 20–30-min recovery between repetitions [15, 16].

#### Speed Endurance

While most scientific studies recommend that sprinting repetitions should be performed with maximal velocity [1–4], acknowledged practitioners have over decades prescribed sprint training during the preparation phase with sub-maximal intensity. Pioneer sprint coach Carlo Vittori (founder of the European School in sprint training and coach of the former 200-m world record holder Pietro Mennea) introduced the “speed endurance” concept already in the mid-1970s [12]. This consisted of series with repeated sprints over 60–80 m, interspersed with approximately 2- and 8-min recovery between sprints and series. The intensity began at 90% of maximal sprint velocity in the initial weeks and progressed to 95% throughout the preparation period. This was accompanied by a gradual increase in total volume from 6 to 800 m (e.g., 2 series of 5 × 60 m) and up to 1500–2000 m (e.g., 5 series of 5 × 60 m) during the preparation phase. However, as the competition season approached, the total volume decreased while the intensity gradually increased to maximal effort [12]. Vittori’s speed endurance concept has later been adopted by other acknowledged sprint coaches [11, 13–16].

Available evidence in endurance and strength training also demonstrates that high but sub-maximal intensity loading effectively stimulates adaptation through the interaction between high intensity and larger accumulated work that can be achieved before the onset of fatigue, compared with maximal efforts [90, 108]. While most practitioners argue that 92–95% intensity is required [11, 13–16], the lowest effective sprinting intensity for stimulating adaptation is so far not established in the research literature. Given the exponential relationship between power and velocity, a reduction from maximal to ~  95% of maximal velocity represents a substantial reduction in force and power load on the neuromuscular system. Most coaches tend to link speed endurance training to the deceleration phase of the sprint. Scientific studies of team sport athletes indicate that sub-maximal sprinting (i.e., ~  90–95% of maximal velocity) is more effective for enhancing maximal velocity than for improving the acceleration phase [109–111].

Practitioners typically assess the athletes’ velocity during sprint training sessions for control and intensity regulation, and timing gates with 10–30-m intervals are typically used for this purpose. The intensity scale in Table 4, which is based on the velocity obtained during 10-, 20-, and 30-m splits (excluding the acceleration phase), can assist practitioners during sprint-specific training sessions.

**Table 4:** Intensity scale for sprint training expressed as 10-, 20-, and 30-m flying splits (s)

Time interval	100%	99%	98%	97%	95%	93%	90%	70%
30-m flying	2.60	2.63	2.65	2.68	2.74	2.80	2.89	3.71
	2.70	2.73	2.76	2.78	2.84	2.90	3.00	3.86
	2.80	2.83	2.86	2.89	2.95	3.01	3.11	4.00
	2.90	2.93	2.96	2.99	3.05	3.12	3.22	4.14
	3.00	3.03	3.06	3.09	3.16	3.23	3.33	4.29
	3.10	3.13	3.16	3.20	3.26	3.33	3.44	4.43
	3.20	3.23	3.27	3.30	3.37	3.44	3.56	4.57
	3.30	3.33	3.37	3.40	3.47	3.55	3.67	4.71
20-m flying	1.73	1.75	1.77	1.79	1.83	1.87	1.93	2.47
	1.80	1.82	1.84	1.85	1.89	1.93	2.00	2.57
	1.87	1.89	1.91	1.93	1.97	2.01	2.07	2.67
	1.93	1.95	1.97	1.99	2.03	2.08	2.15	2.76
	2.00	2.02	2.04	2.06	2.11	2.15	2.22	2.86
	2.07	2.09	2.11	2.13	2.17	2.22	2.29	2.95
	2.13	2.15	2.18	2.20	2.25	2.29	2.37	3.05
	2.20	2.22	2.25	2.27	2.31	2.37	2.45	3.14
10-m flying	0.87	0.88	0.88	0.89	0.91	0.93	0.96	1.24
	0.90	0.91	0.92	0.93	0.95	0.97	1.00	1.29
	0.93	0.94	0.95	0.96	0.98	1.00	1.04	1.33
	0.97	0.98	0.99	1.00	1.02	1.04	1.07	1.38
	1.00	1.01	1.02	1.03	1.05	1.08	1.11	1.43
	1.03	1.04	1.05	1.07	1.09	1.11	1.15	1.48
	1.07	1.08	1.09	1.10	1.12	1.15	1.19	1.52
	1.10	1.11	1.12	1.13	1.16	1.18	1.22	1.57

All calculations are based on the mean velocity obtained during the flying sprints

#### Resisted Sprinting

Resisted sprinting is a commonly used method to overload specific capacities for sprinting acceleration performance, including uphill sprinting, sled sprints, or using motorized devices. Although sled sprints have been most investigated in the research literature [2], uphill sprinting has also been reported as an effective tool for sprint performance improvement, at least in team sport players [112, 113]. It has been suggested that resisted sprint training may be a more effective tool to improve horizontal force and power production during sprinting compared with, e.g., traditional strength and power training performed in the gym [2, 114]. It is hypothesized that better transfer to sprint performance can be achieved if the resistance training exercises mimic the motor pattern and contraction type of performance movement. Resisted sprints are typically categorized based on the performance time decrement induced by the resistance into light (< 10% velocity decrement), moderate (10–15%), heavy (15–30%), and very heavy (> 30%) loads [2]. A limited number of studies have exceeded relatively light resistance loading in fear of constraints such as slower running velocity and/or altered running technique [2, 115]. However, acknowledged scientists have recently questioned this approach, as strength and power exercises with heavy weights might be replaced by moderate to very heavy resisted sprint loading [114, 116, 117]. According to Cross et al. [114], the optimal loading for maximizing power output during resisted sprinting is a resistance that reduces the maximal velocity by ~ 50%. Morin et al. [117] tested the use of very heavy resistance load in soccer players and observed a substantial, increased horizontal force production when compared with non-resisted sprinting. However, only trivial between-group differences were observed for power output and sprint performance. Because peak power output during a maximal sprint is reached after very few steps and falls substantially during the remaining part of the sprint [23, 38], it is reasonable to assume that the entire power output range should be targeted during the training process. What is beneficial for a small portion of the sprint is not necessarily beneficial for overall performance. Haugen et al. [35] proposed that heavy resisted sprinting is likely more appropriate for sports where the athletes are required to perform brief sprints while moving an external mass (e.g., bobsleigh). Overall, the literature is equivocal regarding the potential short-term effects of resisted sprinting when compared with sprinting under normal conditions [2, 3]. Still, specific adaptations are observed for resisted sprint training. That is, resisted sprint training improves resisted sprint performance more than sprint performance under normal conditions [118]. Whether enhanced resisted sprint performance provides potential transfer effects to normal sprinting over time remains unknown.

Resisted sprinting is commonly used in the preparatory training phase among successful sprint groups [10–16]. However, the resistance loading varies across groups and individuals. While the UK Athletics argues that only light loads should be used to ensure proper running mechanics [15, 16], some of the very best Jamaican sprinters (e.g., Asafa Powell) have applied heavy resistance loads during sled sprints [10]. However, resisted sprinting is not prioritized during the competition season in either of these elite sprinting groups.

#### Assisted Sprinting

Assisted sprinting (e.g., downhill running, being pulled by an elastic cord or motorized devices) has occasionally been used by scientists and practitioners as a tool for maximal velocity improvement. Athletes are typically advised to focus on high step rate when approaching their maximal velocity during assisted sprints [103, 119, 120]. That is, supramaximal velocity should be a result of higher step rate, shorter ground contact times, and higher hip angle velocities. Clark et al. [121] observed that towing force magnitude influences the kinematics of supramaximal running. Potentially negative training effects may arise (e.g., increased foot touchdown distance relative to center of mass), and towing force should be individualized to avoid poorer sprint mechanics. Due to the lack of studies investigating assisted sprinting and differences in methodology, it is difficult to draw conclusions from the research literature. Practitioners are generally reluctant to use assisted sprinting devices due to injury risk [10, 11, 13–16], although tail wind sprinting is typically preferred on windy days. Some athletes include assisted sprinting as a part of the warm-up routines prior to competitions. To the best of our knowledge, no studies or practitioners to date have applied assisted sprints for energy preservation purposes. Athletes may be able to perform higher volumes of sub-maximal sprinting (e.g., ~ 95% intensity) during assisted conditions as each sprint is performed with less perceived effort compared to sprinting under normal conditions. This approach remains to be tested.

### Technical Training

Although research literature has emphasized the importance of technique on sprint running performance [20, 24, 33, 38, 40, 49, 51], very few sprint-related studies are devoted to how optimal mechanics can be achieved. The concept of competency-based progression is particularly emphasized in motor learning literature. That is, athletes should not progress to more challenging aspects of training until they master the underpinning principles [122]. Childhood is clearly the most opportune time for fundamental movement skill mastery [123, 124], and acknowledged practitioners have experienced that running movements become more challenging to modify when approaching senior age [10, 11, 15, 16]. Improving a sprinter’s mechanics can be considered a career-long pursuit.

Although sprint training “always” involves technical aspects, sprint drills are commonly used by practitioners to reinforce the technical work, for proprioception, and to isolate specific movement features [10–18]. These include hurdle drills, walking high knees, running high knees, skips, and straight leg bounding, with focus on posture, high hips, front-foot landing, configuration at touchdown and lift-off, etc. Drills are low-speed exercises that are easier to control than high-speed running, typically performed as a part of warm-up routine. Motor learning research tells us that for positive reinforcement of the technique to occur, the biomechanics used in practice must closely resemble those used in competition [89, 122]. Hence, sprint drills must target key technical elements, ensuring crossover effects to normal sprinting over time. Such exercises must be prescribed individually to target the athlete’s limiting factor and provide each athlete a feeling of proper sprinting mechanics [11, 15, 16].

Well-developed coaching skills are a necessity for the practitioner to effectively interact with athletes of all levels [80]. Indeed, coaching communication, feedback, and specific verbal instructions play an integral role in the skill development of sprinting [10, 11, 13, 14]. Although external focus (i.e., on the desired movement effect) has been highlighted in the research literature for enhancing motor performance and skill learning [125–127], most novice coaches use verbal cues during practice that promote an internal focus of attention (i.e., on body movements) [128]. The very best coaches provide allegorical/metaphorical feedback where attention is called upon the athlete’s feeling while executing the practices [129]. For example, the cue “trim the grass with your toes” can be used when the aim is to reduce the flight time during the very first steps of the acceleration phase [128]. Here, art and science do seem to merge, given the interrelation between word choices during instruction, interpreted motor pattern change by athlete, and resulting force and power production. According to Glen Mills, the coach of Usain Bolt, focused athletes with well-developed proprioceptive senses are paramount for coaching to be successful [10].

### Strength and Power Training

Strength and power training has received considerable research attention over the years, and training recommendations for hypertrophy, maximal strength, and power are outlined for novice, intermediate, and advanced athletes [90, 129]. Ballistic exercises with loading up to ~ 60% of one repetition maximum appear to be a highly potent loading stimulus for improving maximal power [90, 130, 131]. However, heavier loading might be necessary to increase the force component of the power equation. Although there is a fundamental relationship between strength and power [130, 132, 133], improvements in sprinting performance do not necessarily occur immediately after a period of strength training [134]. In fact, heavy strength training may induce negative short-term effects on sprint performance [135]. As an athlete gets heavier, the energy cost of accelerating that mass also increases, as does the aerodynamic drag associated with pushing a wider frontal area through the air. “Bigger” is not necessarily better for sprinting, likely explaining why male and female elite sprinters have a body mass of “only” 77 ± 7 and 58 ± 5 kg, respectively [136]. Haugen et al. [35] observed that volleyball/beach volleyball players were among the best sports in terms of horizontal force production during accelerated running, while weight-/powerlifters produced clearly lower values despite no substantial group mean differences in body mass. Vertically oriented and heavy strength training of the lower limbs does not automatically translate to higher horizontal force production during accelerated sprinting [137], but the probability of positive effects increases when strength and sprint training are combined [90, 138, 139].

Strength and power training is crucial parts of the overall training strategy among leading sprint practitioners, and such training is typically performed 2–3 times per week during the preparation period [10, 11, 13, 14, 18]. Exercise selection typically varies from general (e.g., squat, snatch, clean and jerk) to more “sprint specific” (e.g., split squats, single-leg deadlifts, lunges, step-ups, and one-legged squats). Sequencing of sessions differs among coaches, but the majority schedule strength training the day after sprint-specific training to avoid sore muscles when sprinting. Strength and power training is typically structured as consecutive 4–6-week cycles where emphasis is first put on hypertrophy, then maximal strength, and finally explosive strength/power/plyometric training [11, 13, 14, 138]. The goal of this model is to “transform” maximum strength in weight room exercises into functional power on the track. These periods of heavy strength training are often combined with high volumes of sprint training at sub-maximal intensity. The closer to the competition season is, the more emphasis on maximal velocity sprinting, explosive strength, and ballistic exercises [11, 13, 14, 18]. Overall, no major discrepancies in sprint-related strength and power training recommendations can be observed between science and best practice when comparing these literature sources.

### Plyometric Training

Plyometric exercises are characterized by rapid stretch-shortening cycle muscle actions and include a range of unilateral and bilateral bounding, hopping, jumping, and medicine ball throw variations [140]. Plyometric training is normally performed with little or no external resistance and has been shown to significantly improve maximal power output during sport-specific movements [130, 141]. As a rule, the more specific a plyometric exercise is to stretch rate and load characteristics of the sport movement, the greater the transfer of the training effect to performance. Sprinters are encouraged to use different types of high-intensive bounding, jumping, and skipping exercises to ensure that power production is exerted in the horizontal plane [130, 141]. The underlying mechanisms are theorized to elicit specific adaptations in neural drive, rate of neural activation, and intermuscular control, which result in an improved rate of force development [130].

The reutilization of stored energy as a strategy for sprint performance has recently been questioned by Haugen et al. [24], as storage and release of elastic energy take time. Human tendons stretch under load, and sprinters should likely minimize the downside of having these elastic connectors. Adding to the argument, world-class performers sprint with considerably higher leg stiffness than their lower performing counterparts [24]. Based on these considerations, sprinters should focus on leg stiffness (e.g., short ground contact time) during plyometric exercises. Interestingly, this approach was utilized with seeming success by coach Carlo Vittori and the Italian School of sprint training already in the 1970s. The best athlete, Pietro Mennea, performed horizontal jumps and skipping exercises with a weight belt, and ground contact time during these exercises never exceeded 100 ms [12]. This contact time is very similar to those obtained by elite sprinters at maximal velocity [24]. Mennea also performed assisted sprints while equipped with a weight belt (weight vests serve the same purpose). Although these training methods offer strong leg stiffness stimulations, they are demanding and probably increase injury risk, particularly for the Achilles tendon. This may explain why most practitioners perform more traditional plyometric drills as bilateral obstacle (hurdle) jumps, multi jump circuits, medicine ball throws, and unilateral bounding exercises [10–18]. Although the highest volumes are accomplished during the preparation phase, some plyometric training is performed during the competition season [10, 11, 15, 16].

## Recovery Strategies

The performance capacity of an athlete depends on an optimal balance between training and recovery. While sleep and nutrition are fundamental for the restoration of daily life and the recovery process following physical exercise [142–144], several recovery strategies have been explored to improve recovery in athletes. Within leading sprinting communities, so-called tempo runs (100–300 m running with brief recoveries and intensity 60–70% of maximal sprint velocity) are commonly used between days of high-intensive training to loosen up stiff muscles and improve cardiovascular fitness [10, 11, 13–18]. (Note that tempo runs in a sprint training setting are different to those in endurance training settings). Total volume per training session is typically ~  2000 m during the preparation period and ~ 1000 m during the competition period [13–16]. Although the scientific evidence for post-exercise recovery purposes is limited [145–148], tempo runs contribute to a total training volume that may increase the athletes’ trainability and durability in the long term.

A number of passive recovery modalities have also been applied by practitioners over the years, including massage, stretching, compression garments, cold water or contrast water immersion, cryotherapy, hyperbaric oxygen therapy, and electromyostimulation [11, 13, 14]. While there may be some subjective benefits for post-exercise recovery, there is currently no convincing evidence to justify the widespread use of such strategies in competitive athletes [142, 146, 149–162]. Placebo effects may be beneficial, and at the individual level, certain recovery modalities may elicit reproducible acceleration of recovery processes. Future studies of experimental models designed to reflect the circumstances of elite athletes are needed to gain further insights regarding the efficacy of various recovery modalities on sprint performance.

## Tapering

Tapering refers to the marked reduction of total training load in the final days before an important competition. Tapering strategies consist of a short-term balancing act, reducing the cumulative effects of fatigue, but maintaining fitness [163, 164]. Because tapering strategies and outcomes are heavily dependent on the preceding training load, it is often challenging to separate tapering from periodization and training programming in general. According to several authors, a realistic performance goal for the final taper should be a competition performance improvement of about 2–3%. However, these estimates are mainly based on well-trained athletes in endurance- (swimming, running, cycling) or strength-related sports [163–168]. Based on individual performance variation data in elite sprinters [5, 69], it is reasonable to expect smaller relative tapering effects for sprinting athletes.

The general scientific guidelines for a likely effective taper in strength- and power-related sports are a 2- to 3-week period incorporating 40–60% reduction in training volume following a progressive non-linear format, while training intensity and frequency are maintained or only slightly reduced [169–171]. The strategies employed by successful track and field are generally consistent with research [172]. The 10-day taper program developed by Charlie Francis has received considerable attention within the sprinting community [13, 14] (Table 5). Here, provided that the preceding workout the last 6–8 weeks has been performed according to plan (no injuries or disease), the last extensive and high-intensive sprint session is performed 10 days prior to the most important competition of the year, then followed by easy sprint training sessions (low volume at 95% velocity) 8, 6, 4, and 2 days before competition. Stephen Francis argues for a slightly different approach, mainly decreasing the volume by 30% over the last 10 days before a major competition [10]. His most successful athlete, Asafa Powell, achieved world record performances in June as well as September.

**Table 5:** Charlie Francis’ 10-day tapering plan

Days to competition	Training prescription
10 days before	Spikes on track: 4 × 30 m from blocks with full recovery. 80-100-120-150-m flying sprints with maximal intensity, full recovery (i.e., 20–35 min between sprints)
9 days before	Trainers on grass: 10 × 200 m tempo runs with 100-m walking in between
8 days before	Spikes on track: 4 × 30 m from blocks and 1 × 120 m at 95% intensity, full recovery
7 days before	Trainers on grass: 2 × 10 × 100 m tempo runs with 100-m walking in between
6 days before	Spikes on track: 4 × 30 m from blocks and 1 × 150 m at 95% intensity, full recovery
5 days before	No training
4 days before	Spikes on track: 4 × 30 m from blocks and 1 × 80 m at 95% intensity, full recovery
3 days before	Trainers on grass: 10 × 100 m tempo runs with 100-m walking in between
2 days before	Spikes on track: 4 × 30 m from blocks at 95% intensity, full recovery
1 day before	No training

Given that there are several roads to Rome in terms of tapering, it is generally accepted that the training during this period should be highly specific. That is, only exercises that directly assist sports performance should remain, while accessory work and assistance exercises should be removed from the training prescription [169, 171, 172]. Moreover, the number of technical inputs should be kept to a minimum to prepare the athletes mentally and build confidence. Successful coaches adapt a holistic strategy where physiological, technical, and mental aspects are integrated into the tapering process [172]. The individualized approach is consistent with discussions of coaching, reinforcing that not all athletes are the same, nor are circumstances and contexts, and hence, a “one-size-fits-all” approach is rarely appropriate.

## Conclusions

This review has contrasted scientific and best practice literature. Although the scientific literature provides useful and general information regarding the development of sprint performance and underlying determinants, there is a considerable gap between science and best practice in how training principles and methods are applied (these gaps are summarized in Table 6). Possible explanations for these discrepancies may be that scientific studies mainly examine isolated variables under standardized conditions, while best practice is concerned about external validity and apply a more holistic approach. In order to close this gap between science and practice, future investigations should observe and assess elite sprinters throughout the training year, aiming to establish mechanistic connections between training content, changes in performance, and underlying mechanical and physiological determinants. The conclusions drawn in this review may serve as a position statement and provide a point of departure for forthcoming studies regarding sprint training of elite athletic contestants.

**Table 6:** Summary of the level of agreement between scientific and best practice literature

Training principle or method	Scientific versus best practice literature
Progressive overload	*Moderate agreement*. Both scientific and best practice literature emphasize the importance of familiarization and gradual progression to reduce injury risk and maximize performance. However, the influence of running surface and footwear as specific modifiers of sprint training load is more highlighted within best practice.
Specificity	*Poor agreement*. Both scientific and best practice literature highlight the importance of sprint running and high-velocity movements on sprint performance enhancement. However, there is a considerable gap in how the sprint specific training components are applied (see, e.g., specific sprint training further down).
Variation/periodization	*Poor agreement*. Scientific studies mainly focus on traditional and block-training periodization, while alternative models (e.g., “long-to-short” and “short-to-long”) are used within leading sprinting communities.
Individualization	*Poor agreement*. Most scientific interventions have applied a “one-size-fits-all” approach, but recent studies have suggested that training should be prescribed according to individual force-velocity profiles. Best practice focuses more on training prescription according to individual performance capacity, anthropometric factors, training status/age, sex, and recovery/injury status.
Specific sprint training	*Poor agreement*. Most sprint-related studies are performed on young team sport players, consisting of brief and maximal sprints with short recoveries. In contrast, elite sprinters perform sprint-specific training with varying distances, intensities and recoveries.
Technical training	*Poor agreement*. Very few scientific studies are devoted to how optimal sprinting mechanics can be achieved. The best practitioners apply sprint drills to reinforce the technical work and isolate specific movement features.
Strength and power training	*Good agreement*. There are no major discrepancies in sprint-related strength- and power training recommendations when comparing scientific and best practice literature.
Plyometric training	*Good agreement*. Both scientific and best practice literature encourage sprinters to use different types of high-intensive bounding, jumping and skipping exercises for developing leg stiffness and horizontal power production.
Recovery strategies	*Poor agreement*. Best practice applies several passive and active post-exercise recovery modalities (massage, compression garments, cold water immersion, cryotherapy, tempo runs, etc.), although the scientific evidence for these strategies is limited.
Tapering	*Good agreement*. The tapering strategies employed by the best practitioners are generally consistent with research, although best practice literature provides more detailed information.

## Data Availability

Not applicable.
